# Modulating the Release Kinetics of Paclitaxel from Membrane-Covered Stents Using Different Loading Strategies

**DOI:** 10.3390/ma1010025

**Published:** 2008-11-07

**Authors:** Georg Sydow-Plum, Ziyad S. Haidar, Yahye Merhi, Maryam Tabrizian

**Affiliations:** 1 Department of Biomedical Engineering, Faculty of Medicine, McGill University, 3775 Rue University, Lyman Duff Medical Sciences Building, 3^rd^ floor, Montréal (Québec), H3A 2B4, Canada. E-mail: gerog.plum@mcgill.ca (G. S-P.); 2 Center for Biorecognition and Biosensors, McGill University, 3775 Rue University, Lyman Duff Medical Sciences Building, Room 313, Montréal (Québec) H3A 2B4, Canada; 3 Faculty of Dentistry, McGill University, Strathcona Anatomy & Dentistry Building, 3640 Rue University, Montréal (Québec) H3A 2B2 Canada. E-mail: ziyad.haidar@mcgill.ca; 4 Institut de Cardiologie de Montréal, 5000 Rue Belanger Est, Montréal (Québec) H1T 1C8 Canada. E-mail: yahye.merhi@icm-mhi.org

**Keywords:** Chitosan, controlled drug release, hyaluronic acid, polyethylene oxide, Paclitaxel, restenosis, stent, thrombogenecity.

## Abstract

Membrane-covered Express^2TM^ Monorail^®^ stents composed of chitosan (CH) blended with polyethylene oxide (PEO) in 70:30% wt (CH-PEO) were coated with a monolayer of hyaluronic acid (HA). This significantly improved the resistance to platelet adhesion and demonstrated excellent mechanical properties, resisting the harsh conditions during stent crimping and subsequent inflation. CH-PEO/HA membrane was then combined with a paclitaxel (Pac) delivery system via three different approaches for comparison of release profiles of Pac. The activity of Pac in these systems was confirmed since its presence in the membrane significantly decreased cell viability of U937 macrophages. Presented results are promising for applications requiring different release patterns of hydrophobic drugs.

## 1. Introduction

Restenosis is an inflammatory response that may involve thrombus formation causing the re-narrowing of a coronary artery. It occurs within 3 – 6 months in 40 – 50% of patients who have had angioplasty. This incidence was reduced to 20% with the use of bare metal stent implantation procedures, along with the administration of anti-proliferative, anti-migratory and anti-inflammatory drugs [1,2,3]. Consequently, drug-eluting stents (DES) have emerged as a more controlled and effective solution to inhibit the development of in-stent restenosis [4,5,6]. Stents coated with anti-proliferative and anti-inflammatory drugs such as paclitaxel, sirolimus, actinomycin D and tacrolimus acting as inhibitors of cell migration and cell cycle progression are currently under evaluation in several clinical trials [6,7]. Among these drugs, paclitaxel (Pac) has been proven to be one of the most promising agents for the treatment of neo-intimal hyperplasia [7,8]. Pac-eluting stents with the trade name of Taxus^®^ Express^2TM^ from Boston Scientific have received European and FDA approval and are currently used in most stenting procedures [8]. Nonetheless, the well-controlled and effective delivery of Pac remains a challenge. An intact drug-carrying membrane deems necessary for the functionality of the DES and the incorporation of drugs in a biodegradable polymer as a membrane coating has been suggested [9,10]. In a previous work, we have introduced a membrane-covered stent using a blend of chitosan (CH), a natural polysaccharide and polyethylene oxide (PEO) as membrane material [11]. CH is a linear cationic polymer of D-glucosamine obtained by alkaline *N*-deacetylation of chitin that has been extensively studied in recent years [12]. Due to inter- and intra-molecular hydrogen bonding, CH features excellent film forming properties and high mechanical strength suitable for membrane formation [12]. CH matrices of various geometries, pore sizes and orientations can be formed either alone or after blending with various macromolecules such as PEO, a synthetic and neutral water-soluble polymer [12,13]. The permeability of solutes through the membranes prepared by blending CH with PEO is much higher than that through CH alone [14,15]. Therefore, CH-PEO appears to be suitable for improving the permeability of toxic metabolites and reducing thrombogenicity. Amiji *et al*. demonstrated that the permeability coefficient of urea increased from 5.47 × 10^-5^ cm^2^∙min^-1^ in CH to 9.89 × 10^-5^ cm^2^∙min^-1^ in CH-PEO membranes [15]. PEO has also been shown to effectively increase the blood compatibility of polymeric materials including CH [15]. Biomaterials grafted with PEO are able to resist plasma protein adsorption and platelet adhesion predominately by a steric repulsion mechanism [16]. To reduce plasma protein adsorption, platelet adhesion and activation, and thrombus development, the surface of CH membranes can be modified by the electrostatic interaction of cationic CH and anionic polysaccharides such as hyaluronic acid (HA) [11,17]. Drug-eluting membranes consisting of chitosan/hyaluronan multi-layers show promising properties for use as a stent coating material [11,17,18,19,20]. HA is a high molecular weight glycosaminoglycan found in the extracellular matrix of arterial smooth muscle cells and endothelial cells and have shown good blood compatibility [21]. A number of strategies for the modification of HA mainly through the carboxyl and hydroxyl groups have been developed including esterification and carbodiimide chemistry [18,22]. HA has been used to cover stents via cross-linking with *N*-(3-dimethylaminopropyl)-*N*′-ethylcarbodiimide (EDC) leading to a reduced inflammatory response compared to un-coated stainless steel stents in pig coronary arteries [23]. Additionally, it significantly reduced platelet deposition by 55% after 2 hours of blood exposure in exteriorized arteriovenous shunts in baboons [21]. 

**Figure 1:**
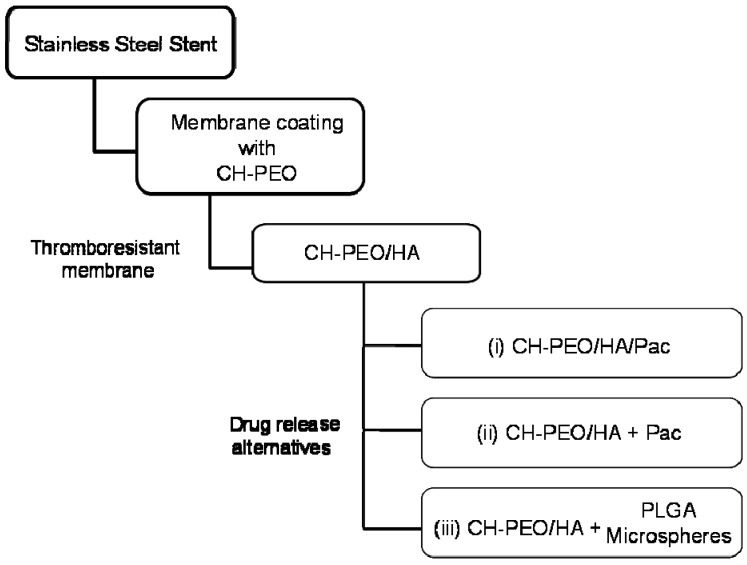
The thromboresistant CH-PEO/HA coating can be used in combination with different alternatives of drug release systems: (i) surface-bound paclitaxel (Pac) via hydrolysable linkage of Pac and HA, (ii) physical incorporation of Pac into the membrane, or (iii) Pac-loaded PLGA microspheres embedded into the membrane.

Many studies have reported the release behaviour of hydrophobic drugs from various polymer systems; however no comparative studies are available showing the effect of the drug loading strategy on release behaviour modulation. In this work, our aim is to investigate and characterize the CH-PEO/HA membrane coating with a drug-eluting membrane system using Pac as a hydrophobic drug model. Initially and as an attempt to improve hemocompatibility, the positively charged CH-PEO membrane was dip-coated with negatively charged HA by means of electrostatic interactions [11,24]. We then investigated the release behaviour of Pac from the CH-PEO/HA membrane where three loading strategies were incorporated (Figure 1). 

In the first system, abbreviated as CH-PEO/HA/Pac, a water-soluble bioconjugate (HA-Pac) was prepared to overcome problems in administering emulsified forms of hydrophobic Pac using carbodiimide coupling chemistry [17] via a labile succinate ester linkage. The layer-by-layer deposition approach between the cationic CH-PEO membrane and the anionic bioconjugate HA-Pac was then applied to form a membrane with surface-bound Pac. In the second strategy marked as CH-PEO/HA+Pac, physical entrapment of Pac inside the membrane was performed as an easy yet effective way to attain a drug delivery system. Finally, in the third strategy, we used a hybrid delivery system consisting of biodegradable poly(D,L-lactic-co-glycolic acid) (PLGA) microspheres [25] incorporated into the membrane as drug vehicles for delivery, and is abbreviated as CH-PEO/HA+PLGA. The membranes were characterized with means of light microscopy and scanning electron microscopy (SEM). Cell viability studies with human macrophages were performed to assess the activity of the released drug with respect to proliferating cells. Thrombogenicity of the membranes was evaluated using ^51^Cr radiolabeled platelet deposition on different CH-based surfaces. Drug release studies for the three systems were carried out in an ethanol/water solution maintaining sink conditions and the released Pac was quantified using HPLC and UV spectrometry and data further fitted in the Higuchi model [26].

## 2. Results and Discussion

### 2.1. CH-PEO/HA membrane characterization 

**Figure 2:**
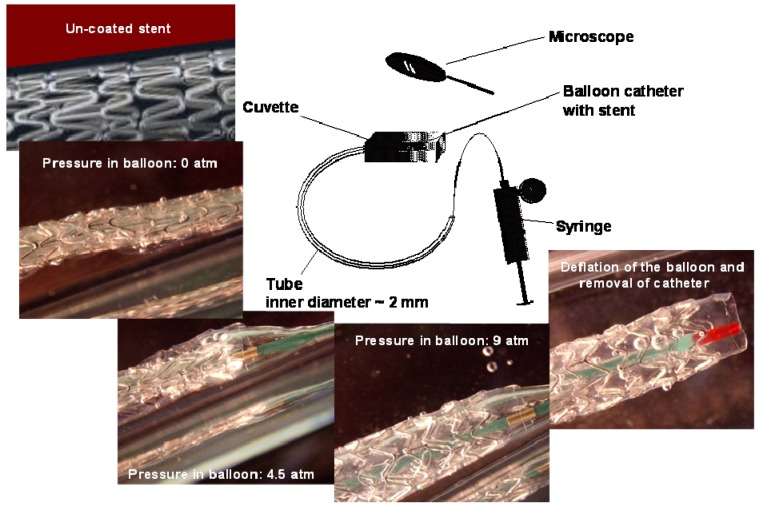
Steps for the inflation of a CH-PEO/HA-covered stent in PBS buffer solution. A quartz cuvette is attached to the end of a tube with an inner diameter of 2 mm to observe the inflation procedure by the microscope.

Figure 2 displays the inflation steps of an Express^2TM^ Monorail^®^ stent system coated with the CH-PEO/HA membrane (inflated diameter 3.0 mm; length 20 mm). The CH-PEO/HA membrane demonstrated excellent mechanical strength resisting the harsh conditions during stent crimping and subsequent inflation in aqueous solution.

**Figure 3:**
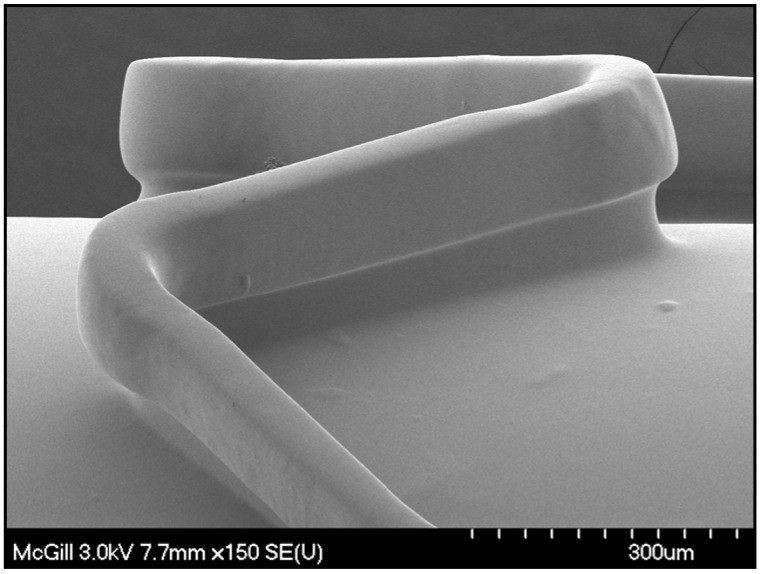
Scanning electron micrograph of an inflated CH-PEO/HA membrane-covered Express^2TM^ stent showing the well-covered stent struts holding the membrane and leading to a tube-like inner surface.

The membrane was intact passing through the tube and could maintain its integrity after undergoing 9 atm pressure. Figure 3 is a scanning electron micrograph of the inflated membrane showing a homogeneous smooth surface without any cracks or damages in the membrane after the above mechanical stress. 

**Figure 4:**
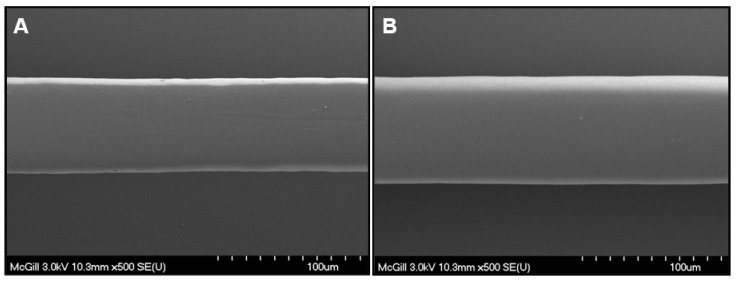
Scanning electron micrograph of an un-coated (A) and CH-PEO/HA membrane-coated (B) Express^2TM^ stent. Strut thickness (A) 65 µm and (B) 76 µm (viewed from above).

A uniformly distributed polymer matrix covers the stent struts while carrying a tube-like membrane formed during inflation. The thickness of the un-coated stent struts and membrane-coated stent struts was determined with SEM (Figure 4). Uncoated struts of Express^2TM^ stents showed a thickness of 65 µm whereas the membrane-coated stents exhibited a thickness of 76 µm, resulting in a dried membrane thickness of only 5.5 µm. Thrombus accumulation was reduced to higher degree on HA coated smooth walled tubes as a result of both surface homogeneity [21] and appropriateness of the membrane thickness [28] which might have significant impact on blood flow and correction of clinical restenosis. It has been shown that the stent struts can be deemed geometrically irregular generating regions of flow re-circulation and stasis, leading to localized blood coagulation, platelet activation, thrombus attachment, and thrombus growth. Conversely, coronary stents with thin struts are associated with a reduced risk for angiographic and clinical restenosis compared to stents with thick struts [28].

### 2.2. Cell viability

**Figure 5:**
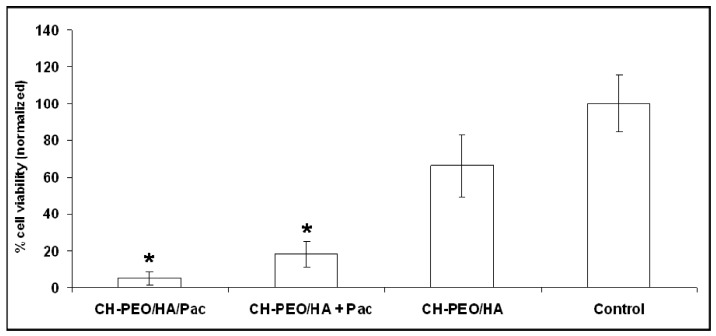
Cell viability of U937 human macrophages determined using Trypan blue assay on different surfaces after 24 hours of exposure time. Cell viability was normalized to the initial number of cells; * indicates p<0.0001, n=5.

The effect of the presence of Pac in the membranes with respect to the cell viability of U937 macrophages is reported in Figure 5. A significant reduction of the cell viability was observed after 24 hours of exposure to respective Pac-containing membranes if compared to the amount of cells at the beginning. Physically-incorporated Pac led to a cell viability of 18.5% ± 6.9% (after 24 hours vs. control, p<0.0001). CH-PEO membranes coated with a monolayer of the bioconjugate HA-Pac led to a cell viability of 5.1 % ± 3.3% (after 24 hours vs. control, p<0.0001). Reduction of cell viability of macrophages was not found to be significant using HA monolayer-coated membranes without Pac (66.2% ± 17.0% after 24 hours vs. cells at the beginning). Even with a low drug concentration as demonstrated with HA-Pac monolayer showed selective toxicity of Pac towards proliferating cells such as U937 macrophages. 

Luo *et al*. [29] suggested that the selective toxicity in case of HA-Pac is due to receptor mediated binding and cellular uptake of the bioconjugate HA-Pac followed by hydrolytic release of the active Pac by cleavage of the labile 2’ester linkage [29]. However, in this study the experiment was different. An uptake of HA-Pac is unlikely because the bioconjugate HA-Pac was attached to the CH-PEO membrane. A cleavage of HA-Pac on the surface of the membrane and diffusion of drug to the cells is more likely in this experimental set-up. Nevertheless, cell viability of proliferating macrophages was reduced indicating the activity of Pac in all cases and their potential in preventing proliferation of smooth muscle cells inside a vessel and thus preventing neo-intimal hyperplasia when applied clinically. 

### 2.3. Paclitaxel Release from CH-PEO/HA membranes

**Figure 6:**
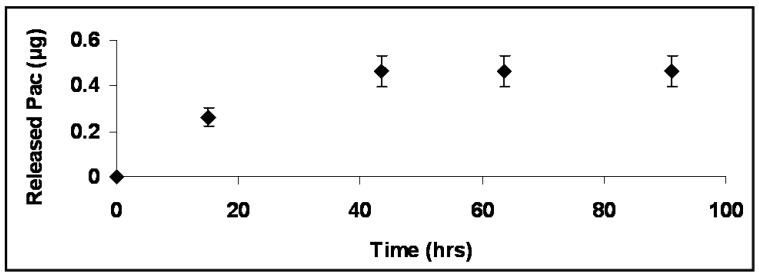
Cumulated release profile of Pac from HA-Pac self-assembled monolayer on a CH-PEO membrane into phosphate-buffered ethanol:water (40:60 v/v) solution (n=3).

*A.* The amount of Pac released from a self assembled monolayer of HA-Pac on the CH/PEO membrane was 0.47 ± 0.07 µg per membrane as shown in Figure 6. After 43 hours, no Pac was detected by HPLC analysis. Thierry *et al*. demonstrated a release of 1.8 µg Pac per cm^2^ constructed by the layer-by-layer technique using substrates with 10 bi-layers of polyelectrolyte HA-Pac(CH/HA-Pac)_9_ [18]. In our study, 0.47 µg Pac per membrane were released corresponding to 0.24 µg per cm^2^. Considering (a) a higher drug loading herein and (b) that results of this study were based on the release of Pac from a monolayer instead of 10 bi-layers, the total amount of released Pac from a monolayer of adsorbed HA-Pac was in agreement with other published data. 

**Figure 7:**
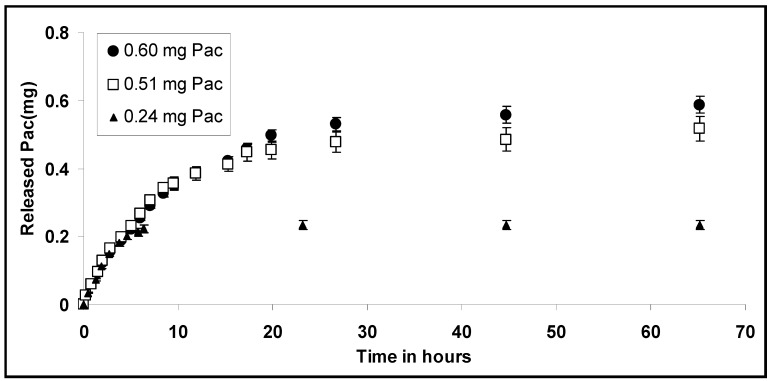
Cumulated release profile of physically incorporated Pac from HA-coated CH-PEO membranes in phosphate-buffered ethanol:water (40:60 v/v) solution. The solvent was exchanged regularly to maintain sink conditions. Three different amounts of Pac (0.24, 0.51 and 0.60 mg) were incorporated (n=3).

*B.* Dried CH-PEO/HA membranes that resulted from 250 µL of 2% CH-PEO solution were loaded with 0.3, 0.7 and 1.0 mg Pac using an ethanolic solution of the drug. The efficiency of drug loading after washing was dependant on the used amount of Pac. Using 0.3, 0.7 and 1.0 mg Pac 80.0 %, 72.8 % and 60.0 % of the drug were finally incorporated in the membrane, respectively (which is 0.24 mg, 0.51 mg and 0.60 mg Pac per membrane in each case). Figure 7 displays the *in vitro* release profiles of Pac from CH-PEO/HA membranes in phosphate-buffered ethanol:water (40:60 v/v) solution. 

The release was observed over a period of 65 hours. *In vitro* release of physically incorporated Pac from CH-PEO/HA membranes showed that 0.233 mg ± 0.014 mg, 0.522 mg ± 0.036 mg and 0.586 mg ± 0.025 mg of Pac was released within 65 hours for 0.24 mg, 0.51 mg and 0.60 mg, respectively. The release of drugs from matrix systems can be described with the Higuchi equation [26]. The basic equation of the Higuchi model is:

(1) $$M_{t}=A\sqrt{Dc_{S}(2c_{0}-c_{S})t}\;\;\text{for}\;{\text{c}}_{0}\;>\;{\text{c}}_{\text{S}}$$

where *M_t_* is the cumulative absolute amount of drug released at time *t*, *A* is the surface area of the controlled release device exposed to the release medium, *c_0_* and *c_S_* are the initial drug concentration, and the solubility of the drug in the polymer, respectively, and *D* is the drug diffusivity in the polymer carrier [26,30]. This model considers diffusion as the dominating mechanism for drug release with the proportionality between the cumulative amount of released drug and the square root of time is an indicator for diffusion controlled drug release systems. The slope *K*, the axis intercept *a* and the squared coefficient of correlation *R^2^* from linear regression were calculated according to equation (2):

(2) $$\frac{M_{t}}{M_{\infty }}=K\sqrt{t}+a$$

where *M_∞_* is the total cumulative amount of drug released at infinite time *t* and *K* is a constant characterizing the design variables of the system [30]. Table 1 illustrates the resulting values from linear regression based on equation (2). A plot of released Pac against the square root of time showed good linearity for all experiments. 

**Table 1:** Slope *K*, axis intercept *a*, squared coefficient of correlation *R^2^* and the plot from equation (2) for the release of physically incorporated Pac (over time in hours) out of CH-PEO/HA membranes in phosphate-buffered ethanol:water (40:60 v/v) solution.

Sample	*K*	*a*	*R^2^*
0.60 mg Pac	0.19	-0.07	0.9959
0.51 mg Pac	0.25	-0.09	0.9956
0.24 mg Pac	0.48	-0.21	0.9959
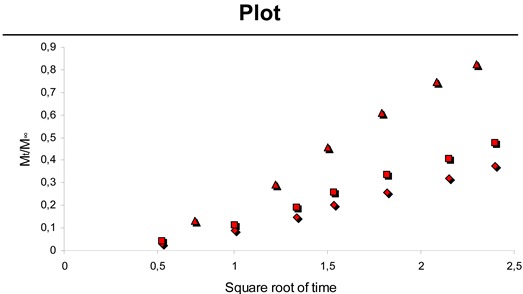			

Negative axis intercepts were attributed to a lag effect particularly occurring when hydrophobic drugs are released into an aqueous medium [31]. Upon incorporating paclitaxel into the hydrophilic CH-PEO/HA membrane a system is obtained, which is able to absorb the ethanol-water solution and to release the entrapped drug by dissolution and diffusion in the gelled matrix, according to Obara *et al*. [32]. Diffusion seems to be the main factor controlling the release of physically incorporated Pac into CH-PEO/HA membranes. 

**Figure 8:**
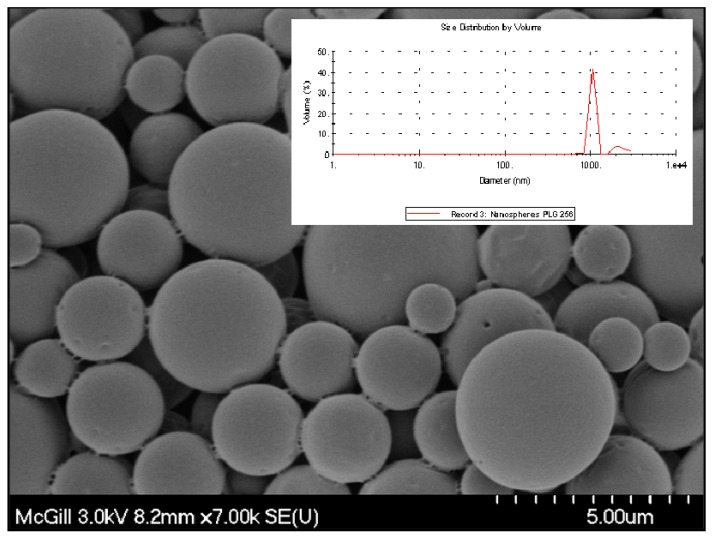
Scanning electron micrograph of PLGA microspheres prepared by the o/w solvent evaporation method. Insert: particle size distribution of the microspheres measured by DLS.

**Figure 9:**
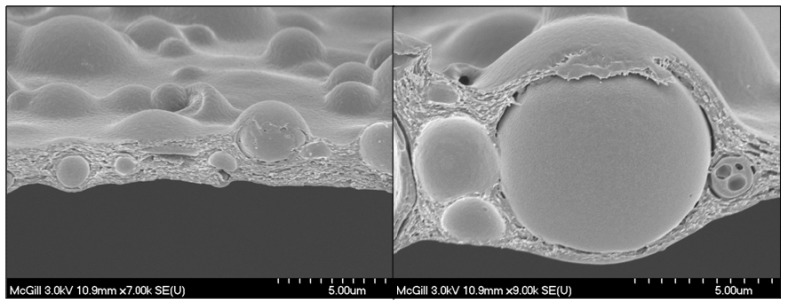
Scanning electron micrographs of a cross section of a CH-PEO membrane with the incorporated PLGA-microspheres.

*C.* For the third system, the CH-PEO/HA membrane with Pac-loaded PLGA, the PLGA microparticles obtained by the o/w solvent evaporation method were spherical in shape and exhibited a smooth surface as shown in Figure 8. Dynamic light scattering (DLS) measurements showed a polydispersity of 0.226 indicating a wider particle size distribution. Additionally, the measured Z-average of 1.29 µm was in a similar range of size found with means of SEM. Figure 9 shows a typical SEM cross-section micrograph of PLGA-microspheres incorporated into CH-PEO membranes with the full coverage of all microspheres evidenced, preventing a wash out of drug-containing particles. 

**Figure 10:**
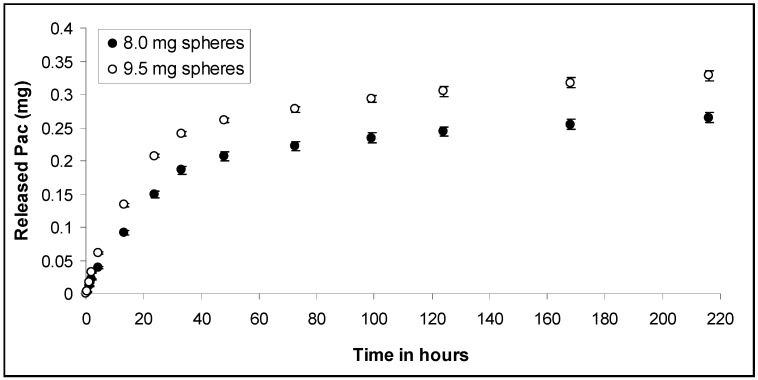
Cumulated release profile of Pac from PLGA-microspheres embedded into CH-PEO/HA membranes in phosphate-buffered ethanol-water (40:60 v/v) solution. Two different amounts of Pac loaded microspheres (0.8 mg and 0.95 mg) were incorporated into the membranes (n=3).

The thickness of the membrane was in the range of 2 to 10 µm. Two different amounts of microspheres with a drug loading of 5.35 % were embedded into the CH-PEO/HA membrane, 8.0 mg and 9.5 mg, respectively. The effect of different incorporation quantity of microspheres into the membrane to the drug release kinetics in phosphate-buffered ethanol:water (40:60 v/v) is illustrated in Figure 10. 

*In vitro* release of Pac from microspheres embedded into CH-PEO/HA membranes showed that 0.264 mg ± 0.008 mg and 0.328 mg ± 0.008 mg of Pac was released within 216 hours for the 8.0 mg and 9.5 mg experiment respectively, which is 61.7 % and 64.6 % of the initially incorporated drug in each case. Table 2 demonstrates the linear regression results based on equation (2) for the release of Pac from microspheres embedded into the CH-PEO/HA membrane. Negative axis intercepts are attributed again to a lag effect particularly occurring when hydrophobic drugs are released into an aqueous medium [31]. 

**Table 2:** Slope *K*, axis intercept *a*, squared coefficient of correlation *R^2^* and the plot from equation (2) for the release of Pac (over time in hours) from microspheres embedded into CH-PEO/HA membranes in phosphate-buffered ethanol:water (40:60 v/v) solution.

Sample	*K*	*a*	*R^2^*
8.0 mg spheres	0.08	-0.056	0.9881
9.5 mg spheres	0.09	-0.060	0.9941
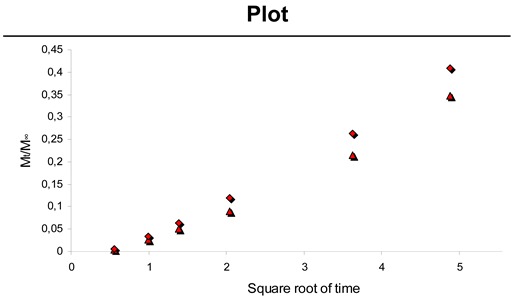			

The calculated squared coefficients of correlation *R^2^* from the release of Pac out of microspheres embedded into the membrane were lower if compared to the release of physically incorporated Pac. The used PLGA polymer for the preparation of microspheres is cleaved into shorter chain alcohols and acids upon contact with water [33]. The Higuchi equation is based on a diffusion controlled system [26,30] and thus polymer degradation contribution to the release mechanism of Pac from PLGA-microspheres explains the lower *K* values in this system if compared to the coefficients found for the release of physically incorporated paclitaxel. The difference in the reported values is not amplified in the release profile due to the variation in the release time. The use of PLGA offers various important advantages over other controlled release systems, such as (a) the possibility to control the release rate over a period of days or months, (b) good biocompatibility and (c) complete biodegradability [33]. PLGA based microparticles are known to be bulk eroding, because water penetration into the system is much faster than the subsequent polymer chain cleavage [33]. The eroding microspheres have to remain in the membrane during degradation to avoid flushing of particles into the bloodstream. Compared to the release of physically incorporated Pac, the drug release from microspheres resulted in significantly slower release. Furthermore, cell culture tests confirmed the activity of low amounts of released Pac from HA-Pac monolayers suggesting the reduction of drug loading for the release of Pac from both, physically incorporated drug into the membranes and Pac-loaded microspheres embedded into the membrane. 

### 2.4. Thrombogenecity

**Figure 11:**
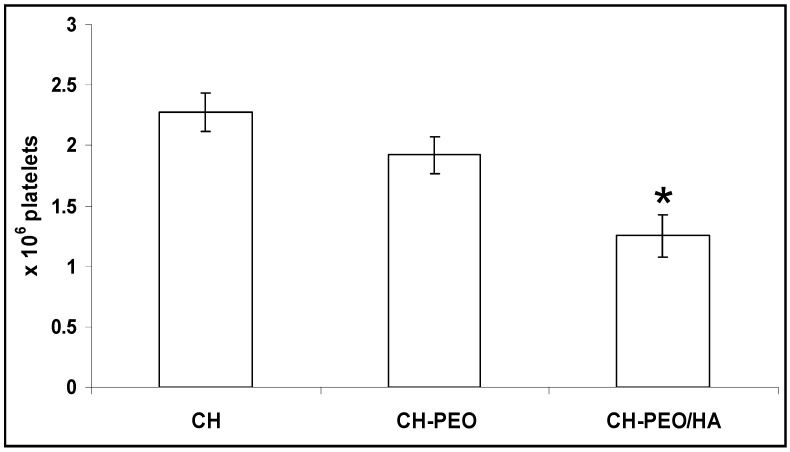
Platelet adhesion on different surfaces: Chitosan (CH), chitosan-PEO blend (CH-PEO), and chitosan-PEO blend coated with a monolayer of hyaluronan (CH-PEO/HA). Pure CH was used as control; * indicates statistical significance with p<0.002, n=3.

Blending CH with PEO improved the resistance to platelet adhesion (2.27 ± 0.16 10^6^ platelets/cm^2^ vs. 1.92 ± 0.15 10^6^ platelets/cm^2^ for CH and CH-PEO respectively), as shown in Figure 11. 

Further improvement was achieved by coating the CH-PEO membrane with a monolayer of HA which led to a significantly reduced platelet adhesion compared to CH alone (1.25 ± 0.17 10^6^ versus 2.27 ± 0.16 10^6^ for CH, p<0.002). Hydrogels are highly hydrated (>95% w/w) and exhibit smooth surfaces counteracting cell attachment [34]. Additionally, recent studies have indicated that the high anionicity of native long chain HA poorly interact with cells in vitro and show poor platelet binding characteristics which is likely due to their extreme hydrophilicity and anionic surface charge. It seems that the surface functional groups of HA and their charge characteristics play an important role in platelet adhesion [34]. 

## 3. Experimental Section

Sodium Hyaluronate (HA) and chitosan (CH: HMW, degree of deacetylation: 85 %), poly(lactic-co-glycolic acid) (PLGA; L:G molar ratio:75:25, MW: 90,000-126,000), polyvinylalcohol (PVA; MW: 50,000-85,000), phosphate buffered saline (PBS), dicyclohexylcarbodiimide (DCC), polyethylene oxide (PEO) (MW: 1,000,000), sodium chlorine, succinic anhydride, ethylenediamine and methylene chlorine (biotech grade) were all purchased from Sigma-Aldrich and used as supplied. 1-(3-Dimethyl-aminopropyl)-3-ethylcarbodiimide (EDC) was obtained from Alfa Aesar (MA, USA) and *N*-hydroxysuccinimide (NHS) was obtained from Fluka (Steinheim, Germany). All media and cells were purchased from HyClone, Logan, UT, USA. Express^2TM^ Monorail^®^ from Boston Scientific (MA, USA) and Paclitaxel were kindly provided by Dr. Luc Bilodeau from the Montreal Heart Institute (Montréal, QC, Canada).

### 3.1. Preparation of hyaluronan (HA) ester prodrug of paclitaxel (Pac)

The HA ester prodrug of Pac (HA-Pac) was prepared in three steps as reported elsewhere [18,25]. Briefly, 2’-Succinyltaxol was prepared and Pac was linked to HA via a labile succinate ester linkage [25]. Subsequently the 2’-succinyltaxol was converted into an activated ester using carbodiimide coupling chemistry with *N*-hydroxysuccinimide (NHS) and dicyclohexylcarbodiimide (DCC). The activated ester was linked to an amine modified HA. The last purification steps in the synthesis of HA-Pac were dialysis of the HA-Pac solution against acetone/water (70:30) and water (membrane tubing, molecular weight cut-off 50000). Pac loading was determined by UV absorbance (λ_max_= 227 nm, ε = 2.8 × 10^4^) in 80:20 CH_3_CN:H_2_O.

### 3.2. CH-PEO membrane formation

CH-PEO blend was used for film preparation and for the membrane-covered stent. CH was dissolved in 0.1 M aqueous acetic acid (2% w/w) and then filtered. PEO dissolved in glacial acetic acid was then added to the CH solution to form a CH/PEO blend with a ratio of 70:30 by weight. The blend was degassed and either used for the formation of films or for the preparation of the membrane covered stent. The dried material was washed with 0.1 N NaOH to de-protonate the ammonium functions of the CH. Subsequently, the membrane was washed thoroughly with PBS buffer and finally with 0.14 M aqueous NaCl. Microsphere-containing membranes were prepared by suspending the microspheres into the CH-PEO blend. All further steps were carried out as mentioned with CH-PEO membrane formation.

### 3.3. Membrane-covered stent preparation

The membrane-covered stent preparation was carried out according to a modified procedure reported by Thierry *et al*. [11]. CH-PEO (70:30) blend solution was used for an Express^2TM^ Monorail^®^ stent system. The stent was mounted on an inflated rotating balloon catheter and the degassed CH-PEO solution was applied onto it. Following overnight drying, the balloon was deflated and the stent was immersed into a 0.1 N NaOH solution. 

### 3.4. Self-assembled monolayer coatings: membrane-HA and membrane HA-Pac preparation

A solution of sodium hyaluronate (1 mg/mL in 0.14 M aqueous NaCl) and a solution of the HA prodrug of paclitaxel (HA-Pac 1 mg/mL in 0.14 M aqueous NaCl) were prepared separately. Ultrapure water (UPW) was used in all experiments (18.2 MΩ cm^2^; MilliQ system, Millipore). The monolayer build-up was accomplished by applying the polymer solution (HA or HA-Pac) onto the CH-PEO substrates. The adsorption time was 5 minutes followed by a washing step with 0.14 M NaCl.

### 3.5. Membrane assessment during inflation

The membrane-covered stent was hand-crimped onto the balloon catheter [Express^2TM^ Monorail^®^ stent system on 5F Guide Catheter/minimum I.D. (0.059″/1.47 mm)]. To simulate the friction generated during the application of a stent via a balloon catheter inside a blood vessel and to assess the membrane integrity during inflation of the CH-PEO/HA covered stent inside the 2mm tube, the procedure was monitored with a microscope through a quartz cuvette which was attached to the tubing. The cuvette and the tube were filled with PBS buffer solution and the inflation was carried out at 37°C. 

### 3.6. Preparation of microspheres

PLGA microspheres were formulated according to the o/w-solvent evaporation method. 1 mL of PLGA solution (50 mg/mL) with 10 % paclitaxel (w/w) in methylene chloride was poured into an aqueous PVA solution (4% w/v, 10 mL). After stirring, the formed emulsion was treated with ultrasound for 5 minutes and subsequently transferred into a 500 mL round bottom flask. The organic phase was removed under reduced pressure. The remaining spheres were centrifuged for 5 min at 345g and the PVA supernatant was removed carefully. The spheres were re-suspended in UPW and ultra-sonicated for 5 minutes. Centrifugation, exchange of the supernatant with UPW and sonication were repeated three times to remove the PVA. The microspheres were dried then by lyophilization. Particle size measurements were carried out using dynamic light scattering (DLS) via a low angle laser light-scattering device (Malvern Instruments HPPS).

### 3.7. SEM analysis

Un-coated and CH-PEO/HA membrane-coated Express^2TM^ stents were sputtered with an ultra-thin layer of Au-Pd. Microscopic imaging using a Field Emission Gun Scanning Electron Microscope (FEGSEM; Hitachi model S-4700) at an accelerating voltage of 5 kV was done.

### 3.8. Determination of drug loading

Drug loading was determined by dissolving accurately weighed amounts of microspheres in dichloromethane and subsequent drug detection at λ_max_= 229 nm (UV µ-Quant, Bio-Tek Instruments, Inc., VT, USA).

### 3.9. Drug activity assessment by cell viability studies

U937 human macrophages were cultured in RPMI 1640 medium (HyClone, Logan UT) supplemented with 5% fetal bovine serum, 100 U/mL penicillin, and 100 µg/mL streptomycin. Macrophages (10^5^ cells/mL) cultured in suspension were exposed to different surfaces. Macrophages alone served as negative control. 100,000 cells per mL were exposed to the CH-PEO/HA membrane, CH-PEO/HA/Pac membrane with chemically linked Pac and to the CH-PEO/HA with physically incorporated Pac. The UV-spectrum of paclitaxel in an ethanol/water solution showed a maximum extinction at λ_max_ = 229nm whereas an aqueous solution of HA did not show any absorption at that range. The synthesized bioconjugate of Pac and HA showed a maximum at 228 nm indicating the successful linkage of Pac to HA. The loading of Pac on the polymer backbone was quantified by UV absorbance at 228 nm. HA-Pac with an amount of 10% Pac was used in all following experiments. Incubations were conducted at 37°C in a humidified 5% CO_2_ environment. After 24 hours the cells were stained with a trypan blue solution and both, the number of stained cells and the total number of cells were counted using a hemocytometer under the microscope. 

### 3.10. Drug release studies 

#### 3.10.1. Drug release study from CH-PEO/HA/Pac membranes 

A 2% CH-PEO solution in 0.1 M acetic acid (250 µL) was cast into 24-well cell culture plates. The solutions were dried over night, de-protonated with 0.1M NaOH, neutralized with PBS-buffer, and subsequently coated with HA-Pac (HA-Pac 1 mg/mL in 0.14M aqueous NaCl). Drug release studies were carried out in phosphate-buffered ethanol-water (40:60 v/v) on a shaking platform under gentle agitation. The solvent was exchanged frequently to maintain sink-conditions. HPLC analytic was used for the quantification of Pac with UV-detection at λ_max_ = 229 nm using acetonitrile water (50:50) (Fisher Scientific, HPLC grade) as mobile phase with a flow rate of 1.2 mL/min. The injection volume was 50 µL. The separation was achieved using a C-18 reverse phase column (LiChrosorp 25 mm, diameter 4.6 mm). The calibration line for Pac in ethanol-water (40:60) showed good linearity (*R^2^* = 0.9996).

#### 3.10.2. Drug release study from CH-PEO/HA + Pac membranes

A 2% CH-PEO solution in 0.1M acetic acid (250 µL) was cast into 24-well cell culture plates. The solutions were dried over night, de-protonated with 0.1 M NaOH, neutralized with PBS-buffer, and subsequently coated with HA (HA 1 mg/mL in 0.14M aqueous NaCl). The dried membranes were loaded with 0.3, 0.7 and 1.0 mg Pac using an ethanolic solution of the drug. After evaporation of ethanol, the dried membranes were treated with 0.5 mL of pure ethanol. After drying membranes were washed with an ethanol-water (40:60 v/v) solution to remove surface-attached Pac. The release experiment of Pac into phosphate-buffered ethanol-water (40:60) solution was carried out on a shaking platform under gentle agitation. The solvent was exchanged frequently to maintain sink conditions. UV analytic was used for the quantification of Pac at λ_max_ = 229 nm. The calibration line for Pac in ethanol:water (40:60) showed good linearity (*R^2^* = 0.9994).

#### 3.10.3. Drug release study from CH-PEO/HA + PLGA membranes

Isolated Pac-loaded microspheres (8.0 and 9.5 mg, respectively) were added to a CH-PEO solution, cast into 24-well cell culture plates and dried over night. The dried membranes were de-protonated with 0.1M NaOH, neutralized with PBS buffer, and subsequently coated with HA (1 mg/mL in 0.14 M aqueous NaCl). Drug release studies were carried out similarly as described earlier. 

### 3.11. Isolation and labelling of platelets

Platelets were isolated and radio-labelled with ^51^Cr as described previously [27]. A human blood sample collected in acid citrate dextrose was centrifuged at low speed to obtain platelet-rich plasma. Incubation with the radioactive ^51^Cr (Amersham International) followed. The suspension was centrifuged to remove unbound ^51^Cr and re-suspended in platelet-poor plasma. 245x10^6^ platelets were found per mL plasma. Platelet adhesion experiments were carried out with different polymer foils and polymer-composites for 1 hour at room temperature. The size of the foils was 1 cm × 0.5 cm resulting in an exposed area of 1 square centimetre (neglecting the height of the foils). Subsequently, the samples were placed in a gamma counter equipped with a nuclide analysing program for ^51^Cr radioactivity determination. The amounts of platelets per square centimetre adhered to the samples was calculated from the samples radioactivity and the cellular count of reference calibration. The calibration line showed a good linearity (*R^2^* = 0.9996) indicating measurement accuracy.

### 3.12. Statistics

Results are expressed as mean values ±standard deviation (SD). Paired student t-tests were used and ρ<0.05 was considered statistically significant.

## 4. Conclusions

In this work, we have reported a membrane-covered stent from CH-PEO blends with attractive mechanical properties and enhanced drug loading and release capacity. We showed that the combination of the hemocompatible CH-PEO/HA membrane with a drug release system is feasible and that drug release kinetics can be modulated using the desired loading strategy. The absence of surface irregularities due to the HA coating shows the potential for abolishing platelet deposition and thus thrombus formation. The membrane offers the possibility of incorporating the anti-proliferative agent paclitaxel using various strategies: (i) surface bound paclitaxel via hydrolysable ester linkage of drug and HA, (ii) physical incorporation of paclitaxel into the membrane, and (iii) PLGA microspheres loaded with paclitaxel and embedded into the membrane to modulate drug release. Significantly slower release of paclitaxel from PLGA microspheres (~ 60% in 216 hours) could be achieved if compared with the release from physically incorporated or covalently-bound paclitaxel (~ 100% in 65 and 43 hours, respectively). In either membrane system, the Pac-loaded membranes inhibit the viability of proliferating cells such as U937 human macrophages as a result of drug release and demonstrate improved thrombogenicity due to a monolayer coating of the membrane with HA. Thus, it is promising for the clinical administration of highly hydrophobic drugs to more than one application requiring different release patterns such as in the treatment of aneurysms as well as in rare and life-threatening complications of sealing coronary perforations. 

## References

[1] Dobesh P.P., Stacy Z.A., Ansara A.J., Enders J.M. (2004). Drug-eluting stents: A mechanical and pharmacologic approach to coronary artery disease. Pharmacotherapy.

[2] Serruys P.W., Kutryk M.J., Ong A.T. (2006). Coronary-artery stents. N. Engl. J. Med..

[3] Wenaweser P., Daemen J., Zwahlen M., van Domburg R., Jüni P., Vaina S., Hellige G., Tsuchida K., Morger C., Boersma E., Kukreja N., Meier B., Serruys P.W., Windecker S. (2008). Incidence and correlates of drug-eluting stent thrombosis in routine clinical practice. 4-year results from a large 2-institutional cohort study. J. Am. Coll. Cardiol..

[4] Plum G., Tabrizian M. (2005). Clinical Outcomes of Non Pharmaceutical Stent Coatings. Fundamentals About Stents; Proceedings of the European Symposium of Vascular Biomaterials.

[5] Menown I., Lowe R., Penn I. (2005). Passive stent coatings in the drug-eluting era. J. Invasive Cardiol..

[6] Presbitero P., Asioli M. (2002). Drug-eluting stents do they make the difference?. Minerva Cardioangiol..

[7] Billesfeld L., Gerckens U., Muller R., Grube E. (2003). Long-term evaluation of paclitaxel-coated stents for treatment of native coronary lesions. First results of both the clinical and angiographic 18 month follow-up of TAXUS I. Z. Kardiol..

[8] Aoki J., Mintz G.S., Weissman N.J., Mandinov L., Grube E., Dawkins K.D., Ellis S.G., Greenberg J., Yu A., Mann J.T., Cannon L., Cambier P.A., Stone G.W. (2008). Impact of mild or moderate renal insufficiency on the intravascular ultrasonic analysis of chronic vascular response to paclitaxel-eluting and bare-metal stents (from the TAXUS IV, V, and VI trials). Am. J. Cardiol..

[9] Mehvar R. (2003). Recent trends in the use of polysaccharides for improved delivery of therapeutic agents: pharmacokinetic and pharmacodynamic perspectives. Curr. Pharm. Biotechnol..

[10] Prabaharan M., Mano J.F. (2005). Chitosan-based particles as controlled drug delivery systems. Drug Deliv..

[11] Thierry B., Merhi Y., Silver J., Tabrizian M. (2005). Biodegradable membrane-covered stent from chitosan-based polymers. J. Biomed. Mater. Res. A..

[12] Chandy T., Sharma C.P. (1990). Chitosan-as a biomaterial. Biomater. Artif. Cells Artif. Organs..

[13] Budtova T., Belnikevich N., Kalyuzhnaya L., Alexeev V., Bronnikov S., Vesnebolotskkaya S., Zoolshoev Z. (2002). Chitosan modified by poly(ethylene oxide): Film and mixture properties. J. Appl. Polym. Sci..

[14] Neto C.G., Dantas T.N., Fonseca J.L., Pereira M.R. (2005). Permeability studies in chitosan membranes. Effects of crosslinking and poly(ethylene oxide) addition. Carbohydr. Res..

[15] Amiji M. (1995). Permeability and blood compatibility properties of chitosan-poly(ethylene oxide) blend membranes for haemodialysis. Biomaterials.

[16] Amiji M., Park K. (1993). Surface modification of polymeric biomaterials with poly(ethylene oxide), albumin, and heparin for reduced thrombogenicity. J. Biomater. Sci. Polym. Ed..

[17] Thierry B., Winnik F.M., Merhi Y., Silver J., Tabrizian M. (2003). Bioactive coatings of endovascular stents based on polyelectrolyte multilayers. Biomacromolecules.

[18] Thierry B., Kujawa P., Tkaczyk C., Winnik F.M., Bilodeau L., Tabrizian M. (2005). Delivery platform for hydrophobic drugs: prodrug approach combined with self-assembled multilayers. J. Am. Chem. Soc..

[19] Yang Y., Moussa I. (2005). Percutaneous coronary intervention and drug-eluting stents. Can. Med. Ass. J..

[20] Schneider A., Vodouhe C., Richert L., Francius G., Le Guen E., Schaaf P., Voegel J.C., Frisch B., Picart C. (2007). Multifunctional polyelectrolyte multilayer films: Combining mechanical resistance, biodegradability, and bioactivity. Biomacromolecules.

[21] Verheye S., Markou C.P., Salame M.Y., Wan B., King S.B., Robinson K.A., Chronos N.A., Hanson S.R. (2000). Reduced thrombus formation by hyaluronic acid coating of endovascular devices. Arterioscler. Thromb. Vasc. Biol..

[22] Hahn S.K., Hoffman A.S. (2005). Preparation and characterization of biocompatible polyelectrolyte complex multilayer of hyaluronic acid and poly-l-lysine. Int. J. Biol. Macromol..

[23] Heublein B., Evagorou E.G., Rohde R., Ohse S., Meliss R.R., Barlach S., Haverich A. (2002). Polymerized degradable hyaluronan--a platform for stent coating with inherent inhibitory effects on neointimal formation in a porcine coronary model. Int. J. Artif. Organs.

[24] Lu H., Hu N. (2006). Loading behavior of (chitoan/hyaluronic acid)n layer-by-layer assembly films toward myoglobin: an electrochemical study. J. Phys. Chem. B.

[25] Deutsch H.M., Glinski J.A., Hernandez M., Haugwitz R.D., Narayanan V.L., Suffness M., Zalkow M.L. (1989). Synthesis of congeners and prodrugs. 3. Water-soluble prodrugs of taxol with potent antitumor activity. J. Med. Chem..

[26] Higuchi T. (1961). Rate of release of medicaments from ointment bases containing drugs in suspension. J. Pharm. Sci..

[27] Bienvenu J.G., Tanguay J.F., Chauvet P., Merhi Y. (2001). Relationship between platelets and neutrophil adhesion and neointimal growth after repeated arterial wall injury induced by angioplasty in pigs. J. Vasc. Res..

[28] Kastrati A., Mehilli J., Dirschinger J., Dotzer F., Schuhlen H., Neumann F.J., Fleckenstein M., Pfafferott C., Seyfarth M., Schomig A. (2001). Intracoronary stenting and angiographic results: Strut thickness effect on restenosis outcome (ISAR-STEREO) trial. Circulation.

[29] Luo Y., Ziebell M.R., Prestwich G.D. (2000). A hyaluronic acid-taxol antitumor bioconjugate targeted to cancer cells. Biomacromolecules.

[30] Siepmann J., Peppas N.A. (2001). Modeling of drug release from delivery systems based on hydroxypropyl methylcellulose (HPMC). Adv. Drug Deliv. Rev..

[31] Plum G. (2004). *Freisetzungsverhalten von unterschiedlich hydrophil/hydrophoben Modellsubstanzen aus Poly-(D,L-lactid)*- *und Poly-(D,L-lactid)-co-PEO-co-poly-(D,L-lactid)-Mikrosphären und pharmazeutische Anwendungen*. Thesis.

[32] Obara K., Ishihara M., Ozeki Y., Ishizuka T., Hayashi T., Nakamura S., Saito Y., Yura H., Matsui T., Hattori H., Takase B., Ishihara M., Kikuchi M., Maehara T. (2005). Controlled release of paclitaxel from photocrosslinked chitosan hydrogels and its subsequent effect on subcutaneous tumor growth in mice. J. Contr. Release..

[33] Siepmann J., Elkharraz K., Siepmann F., Klose D. (2005). How autocatalysis accelerates drug release from PLGA-based microparticles: a quantitative treatment. Biomacromolecules.

[34] Amarnath L.P., Srinivas A., Ramamurthi A. (2006). *In vitro* hemocompatibility testing of UV-modified hyaluronan hydrogels. Biomaterials.

